# Integrating High throughput Sequencing into Survey Design Reveals Turnip Yellows Virus and Soybean Dwarf Virus in Pea (*Pisum Sativum*) in the United Kingdom

**DOI:** 10.3390/v13122530

**Published:** 2021-12-16

**Authors:** Aimee R. Fowkes, Sam McGreig, Hollie Pufal, Shona Duffy, Becky Howard, Ian P. Adams, Roy Macarthur, Rebecca Weekes, Adrian Fox

**Affiliations:** 1 Fera Science Ltd., Sand Hutton, York YO41 1LZ, UK; Sam.McGreig@fera.co.uk (S.M.); Ian.Adams@fera.co.uk (I.P.A.); Roy.Macarthur@fera.co.uk (R.M.); Rebecca.Weekes@fera.co.uk (R.W.); Adrian.Fox@fera.co.uk (A.F.); 2 School of Natural and Environmental Sciences, University of Newcastle, Newcastle NE1 7RU, UK; h.l.pufal2@newcastle.ac.uk; 3 Processors & Growers Research Organisation (PGRO), Peterborough PE8 6HJ, UK; Shona@pgro.org (S.D.); Becky@pgro.org (B.H.)

**Keywords:** pea viruses, high throughput sequencing, surveillance

## Abstract

There is only limited knowledge of the presence and incidence of viruses in peas within the United Kingdom, therefore high-throughput sequencing (HTS) in combination with a bulk sampling strategy and targeted testing was used to determine the virome in cultivated pea crops. Bulks of 120 leaves collected from twenty fields from around the UK were initially tested by HTS, and presence and incidence of virus was then determined using specific real-time reverse-transcription PCR assays by testing smaller mixed-bulk size samples. This study presents the first finding of turnip yellows virus (TuYV) in peas in the UK and the first finding of soybean dwarf virus (SbDV) in the UK. While TuYV was not previously known to be present in UK peas, it was found in 13 of the 20 sites tested and was present at incidences up to 100%. Pea enation mosaic virus-1, pea enation mosaic virus-2, pea seed-borne mosaic virus, bean yellow mosaic virus, pea enation mosaic virus satellite RNA and turnip yellows virus associated RNA were also identified by HTS. Additionally, a subset of bulked samples were re-sequenced at greater depth to ascertain whether the relatively low depth of sequencing had missed any infections. In each case the same viruses were identified as had been identified using the lower sequencing depth. Sequencing of an isolate of pea seed-borne mosaic virus from 2007 also revealed the presence of TuYV and SbDV, showing that both viruses have been present in the UK for at least a decade, and represents the earliest whole genome of SbDV from Europe. This study demonstrates the potential of HTS to be used as a surveillance tool, or for crop-specific field survey, using a bulk sampling strategy combined with HTS and targeted diagnostics to indicate both presence and incidence of viruses in a crop.

## 1. Introduction

Peas are a key horticultural crop with produce for human consumption as fresh or frozen and combinable peas for the production of pea protein e.g., cattle feed. Peas are also a key element in crop rotations for nitrogen fixation [1]. In the UK, fresh peas and beans have a planted area of approximately 40,000 hectares per year and the planted area of combinable peas for dried pea production has increased approximately 25% between 2018 and 2020 to a planted area of 52,000 hectares [2].

Viruses reported from pea crops in the United Kingdom (UK) largely originate from pre-1960s research [1]. Seven viruses have been reported infecting UK pea crops, but a further 20 viruses are recorded as present in the UK and are known to infect peas but have not been previously reported in peas in the UK.

Conventional approaches to conducting virus surveys tend to focus on collecting multiple symptomatic samples which get tested for a set number of target viruses, using targeted approaches such as ELISA or PCR, the suite of viruses would likely comprise those viruses expected to be present and those viruses known to infect the host which have not yet been detected in a given geographic region. Where there is limited knowledge of the presence of a disease in a crop, a diagnostic focus on the viruses expected to be present, may lead to the exclusion of potentially important pathogens which may not previously have been reported in that region or host, or may be as yet undescribed [3,4]. Due to the high number of potential targets to include in a screen for viruses, where to prioritise diagnostic resource to get the ‘best’ survey presents a challenge. Additionally, focussing solely on symptomatic individuals in a crop would risk missing asymptomatic infections (i.e., those with masked infections, or those individuals carrying early stage infection which may later develop symptoms). Determining the infection status of asymptomatic individuals can also later inform risk assessment or determination of symptom causation [3].

High-throughput sequencing (HTS) is now being widely applied in plant pathology to identify putative viral sequences which are then subsequently confirmed using conventional targeted methods. Currently, most applications of HTS have focussed on resolving the causal agents of diseases of unknown aetiology e.g., [5,6,7] and to determine the presence of novel viruses in biosecurity applications [4]. It is also providing key insights into virus population structure, virus ecology and evolution [8,9,10]. Within regulatory plant health, broad uptake of HTS has been slow due to the constraints of generating validation data to cover using the technology, and also in dealing with assessing the risks of previously undescribed pathogens [8,9,11,12]. There are an increasing number of ways HTS could be used in plant health, such as targeted detection of viruses for germplasm certification [13] or for the surveillance of cultivated and weed hosts for viruses [14,15,16,17,18,19,20,21] and bacterial/fungal pathogens [22]. These studies can provide information on not only what disease are present but can inform the genotypes present which can help understand virus origin and is useful for improving diagnostic testing. A baseline of diseases present can help to inform management strategies. HTS within a national survey has been previously discussed [12]. However, whilst these approaches are currently being investigated, there are no studies looking how HTS could be combined with conventional diagnostic approaches and integrated into a crop-specific survey programme. Han et al. [18] and Gaafar et al. [17] both used HTS for the assessment of viruses in legumes in Ohio and Germany, respectively. Han et al. [18] preferentially collected symptomatic soybean plants and Gaafar et al. [17] collected symptomatic and asymptomatic pea plants, surrounding legume plants and non-legume plants. Virus presence was confirmed by using PCR. Both studies identified both known and novel viruses in the crop of interest, they also determined viruses of concern and the virus distribution. However, whilst these studies indicate the presence and distribution of pathogens they did not clearly elucidate the incidence of the pathogens across a range of sites, which is essential data for understanding the dynamics of plant pathogen epidemics and supporting pest risk analysis.

Therefore, the aim of this study was both to identify what viruses are present in UK peas and at what incidence. The approach detailed below uses a standardised random sampling strategy to take a sample to represent the field, these leaves were then sub-sampled into both a ‘bulked field sample’ (BFS) and smaller bulks of mixed sizes. The whole crop sample was tested by HTS and resulting sequence data was then used to infer the presence of candidate virus targets. Subsequent testing used mixed-bulk size sub-samples and real-time reverse-transcription PCR (real-time RT-PCR), to both confirm the presence of inferred viruses and to estimate the prevalence of those viruses in plants in field samples. Due to the relatively low depth of sequencing used to generate candidate virus sequences, a sub-set of ‘bulk field’ pooled samples were reanalysed by sequencing at increased depth to investigate the validity of the pooled sequencing approach. Additionally, this approach was accompanied by testing a limited number of symptomatic samples to compare the two approaches for efficacy of approach.

## 2. Materials and Methods

### 2.1. Field Sampling

**Figure 1:**
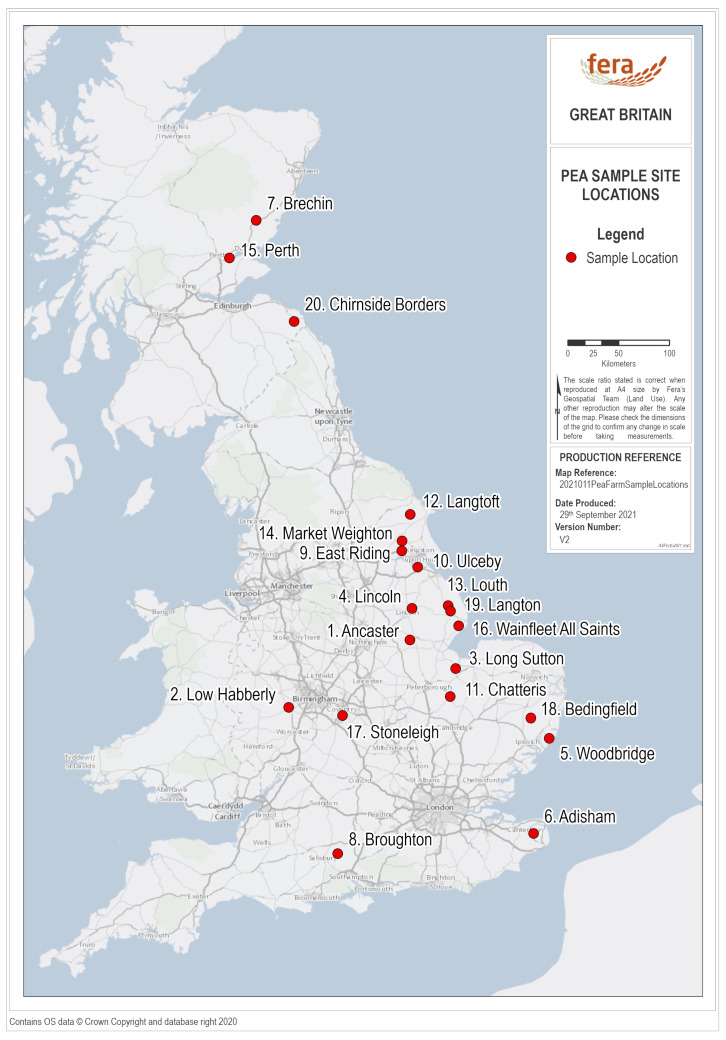
Map of mainland United Kingdom showing the approximate locations of the twenty pea field sites chosen for sampling, made using ArcGIS Pro.

Between May and July 2019, twenty pea crops from around UK were sampled by staff from Processors and Growers Research Organisation (PGRO) (Figure 1). Sample sites were chosen to give a broad geographic spread across the country, but also to reflect the distribution of pea crops in the UK, with a higher density of crops, and consequently sample sites, in the East of the country and Midlands/North of England.

At each field, a 100 m^2^ square was randomly selected. From this eleven parallel transect-lines, at 10 m intervals were made and a single plant sampled at random at each 10 m interval along each transect, making a total of 121 sample points [23]. Each individual plant was sampled by taking the top two open leaves. All plant samples from a field were then bulked together and samples were then sent to Fera Science Ltd. for virus analysis.

**Figure 2:**
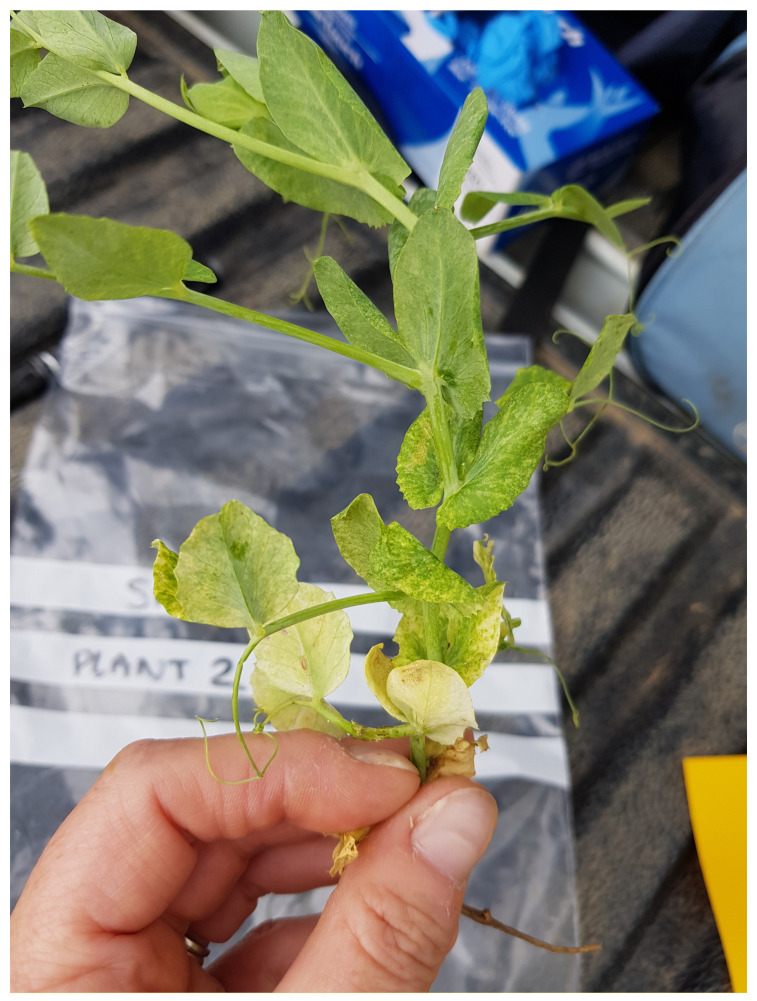
Symptomatic pea plant from site 19 showing mosaic symptoms.

Additionally, 28 individual plants exhibiting symptoms consistent with viral infections, such as yellowing, mosaic and thickening of leaves (See Figure 2), were screened by HTS. Of these, 19 originated from sites included in the bulk sample survey but occurred outside the sampled area. A further nine samples were submitted from sites not included in the field survey.

### 2.2. Sub-Sampling for Virus Analysis

**Figure 3:**
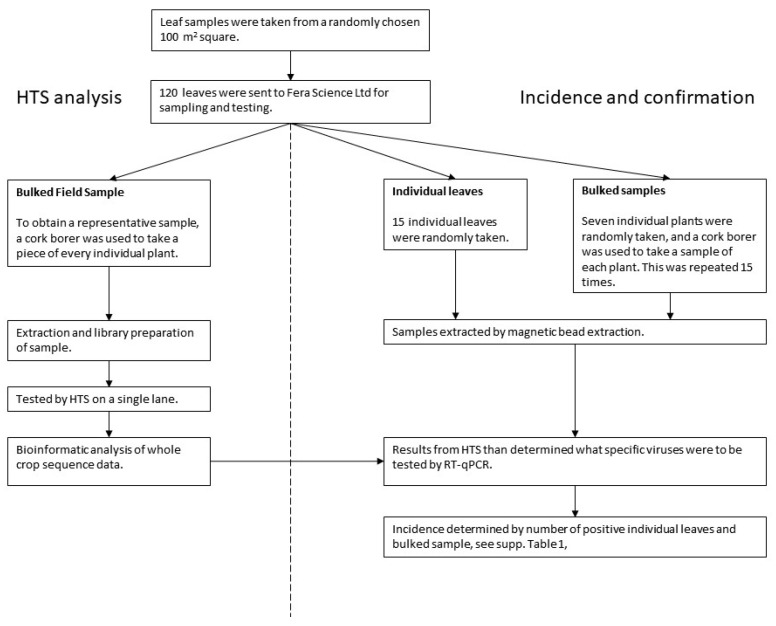
Pea sampling and testing flow chart.

The process used to analyse the pea leaf samples collected at the twenty sites is shown in Figure 3. The flow chart shows how the 120 leaves collected in the field were sampled for analysis by HTS (Bulked Field Sample–BFS) was used to inform testing of multiple sub-samples from the BFS by real-time RT-PCR to confirm the presence of, and determine the incidence of, selected candidate viruses. The details of the sampling and testing is given below.

From the bulked field sample 120 leaves were selected and then sampled in two ways:

(a) HTS analysis–Bulk field sample (BFS): A representative sample of the whole field was obtained by using a cork borer to take a piece of every leaf. This bulk field sample was tested by HTS.
(b) Confirmation and incidence–Individual leaves and bulked samples: Subsamples were also taken from the initial field sample for both confirmation of HTS detections and estimating the incidence of viruses. (i) Fifteen individual leaves were randomly chosen, and (ii) fifteen bulked samples of seven leaves were randomly chosen. A cork-borer was used to obtain a piece of each leaf within the bulked sample.

The additional symptomatic samples from the BFS-sample sites were analysed as four bulks, representing symptomatic samples from sites Market Weighton, Wainfleet, Eye and Langton, and these bulks were run on the MiSeq. The nine general surveillance samples were run individually on the MiSeq. Where possible, symptomatic material was taken. If no symptoms were present, material was taken from around the plant.

### 2.3. Historical Isolate

A pea sample held in the Fera Freeze-Dried Virus isolate collection was tested by HTS as part of ongoing work to characterise the viruses in the collection. The sample originated from East Anglia, United Kingdom and was sent in to Fera Science Ltd. for testing in 2007. The sample was originally screened for pea enation mosaic virus-1 (PEMV-1), pea early browning virus (PEBV) and pea seed-borne mosaic virus (PSbMV) by ELISA. The sample tested positive for PSbMV and was freeze-dried as a reference isolate of the virus. In 2019 the sample was tested by HTS and real-time RT-PCR and RT-PCR as described below.

### 2.4. HTS–RNA Extraction, Library Preparation and Analysis

The BFS, bulked symptomatic samples and individual symptomatic samples were extracted by Qiagen RNeasy mini kit with a DNase step, following manufacturers’ instructions. The RNA extract was ribosome depleted using the Ribozero plant leaf kit (Illumina, San Diego, CA, USA) and dual unique indexed libraries produced using the TruSeq stranded RNA library prep kit (Illumina) as per the manufacturer’s instruction. The resulting BFS libraries were pooled with other indexed libraries, diluted to 10 pM, mixed with 5% PhiX library (Illumina) and sequenced on an Illumina MiSeq using a 600 cycle V3 kit. The resulting sequences were analysed using the “ANGUA” pipeline briefly as follows: Sequences were trimmed to remove low-quality nucleotides from the 3′ end, using a Phred score threshold of 20 using Sickle [24], and assembled using Trinity [25]. Contigs produced were then filtered into two subsets depending on contig length. Contigs with a length equal to or greater than 200 nucleotides (nt) were subject to a BLASTn+ search against the complete NCBI GenBank nt database, and contigs with a length equal to or greater than 1000 nt were subject to a BLASTx+ search against the complete NCBI GenBank nr database [26]. Viral reads were then extracted using MEGAN community edition [27].

The described pipeline is maintained within a Conda environment, allowing for easy software version control, environment portability and program management. From within this environment, the Angua2 script can be run, which automates directory structure, trimming, assembling, contig filtering, BLASTx/BLASTn execution and MEGAN file creation. An experimental version of this pipeline, along with the Conda environment setup file, is publicly available and can be found here: https://fred.fera.co.uk/smcgreig/angua (accessed on 17 November 2021).

Phylogenetic trees were produced using the Maximum likelihood algorithm and 500 bootstraps in MEGA 7 [28]. Pairwise identities were calculated using the same software.

### 2.5. Sub-Sample RNA Extraction, RT-qPCR, and RT-PCR Confirmation

For virus confirmation and incidence testing, individual leaves, bulked samples and symptomatic plants were extracted by a magnetic bead extraction, using Invimag Virus RNA/DNA mini-kit (Invitek GmbH, Berlin, Germany). The samples were then tested for the internal control Cox [29], and subsequently by specific real-time RT-PCR for targets identified by HTS. All assays used are listed in Table 1. The real-time RT-PCR assays for pea enation mosaic virus-1, pea enation mosaic virus-2 and pea seed-borne mosaic virus were designed for this study. Primers and probes were designed using PRIMER EXPRESS v.2 (LifeTech, Shenzhen, China) using sequences available on GenBank.

The detection of soybean dwarf virus (SbDV) was confirmed by first testing by real time RT-PCR, samples which tested positive were then tested by reverse-transcriptase PCR and sent for Sanger sequencing (MWG GmbH, Osterwieck, Germany) [30,32], assays listed in Table 1.

Real-time RT-PCR assay were carried out using 10 µL 2× iTaq universal probes reaction mix (Biorad, Hercules, CA, USA), 0.05 µL iScript reverse transcriptase (Biorad), 1 µL of 7.5 µM forward and reverse primers, 0.5 µL of 5 µM probe, 1 µL sample and molecular grade water to make a total volume of 20 µL. Samples were cycled at 50 °C for 10 min, 95 °C for 2 min, followed by 40 cycles of 95 °C for 15 s and 60 °C for 1 min. Assays were carried out on either 7900HT Fast Real-Time PCR System, 7500 Real-Time PCR System, ViiA 7 Real-Time PCR System, QuantStudio 6 Real-Time PCR System or QuantStudio 12K Real-Time PCR System (Applied Biosystems, Waltham, MA, USA). Preliminary validation of assays indicated a cut off of Ct 35 was suitable for determining a positive detection (data not presented). Any Ct higher than this was recorded as negative.

RT-PCR was carried out using 12.5 µL 2× 1-Step PCR Reddymix (ThermoFisher, Waltham, MA, USA), 1 µL Verso enzyme mix, 9 µL molecular grade water, 1 µL of 10 µM forward and reverse primers and 1 µL sample. Samples were cycled at 50 °C for 15 min, 95 °C for 2 min, followed by 35 cycles of 95 °C for 30 s, 55 °C for 30 s, 72 °C for 1 min, and final elongation of 72 °C for 5 min. RT-PCR products were analysed by electrophoresis using 1% agarose gel stained with ethidium bromide.

### 2.6. Estimating Prevalence Based on Tests Using Different Pool Sizes

Hepworth [33] describes a method for estimating prevalence in a population and the size of the uncertainty about that estimate where samples taken from randomly selected individuals are put into pools, which may be of different size. Within this study this has been implemented in an R [34] function (bgtvs in the package BinGroup version 2.2-1 [35]). Lookup tables were created to provide estimates of virus prevalence for results that may be produced by the testing of pools. Two lookup tables were produced: one for testing 120 plants in 30 pools of 4 plants each, and one for testing 120 plants in 15 pools of 7 plants and 15 individual plants (Tables S3 and S4). The tables were produced by running the bgtvs function for 0 to 30 positive 4-plant pools and for all 225 possible combinations of 0 to 15 positive pools in each of the 15 7-plant and 15 1-plant pools. Finally, combinations of test results which were very unlikely to occur (those where the number of positives among single-plant pools are high and the number of positives among 7-plant pools are low) were removed from the table. This was done where the probability of observing a number of positive pools at or below the observed number of single-plant pools, or at or above the observed number of seven plant pools was less than 0.001 (1 in 1000).

### 2.7. HTS–Increased Depth Testing

**Figure 4:**
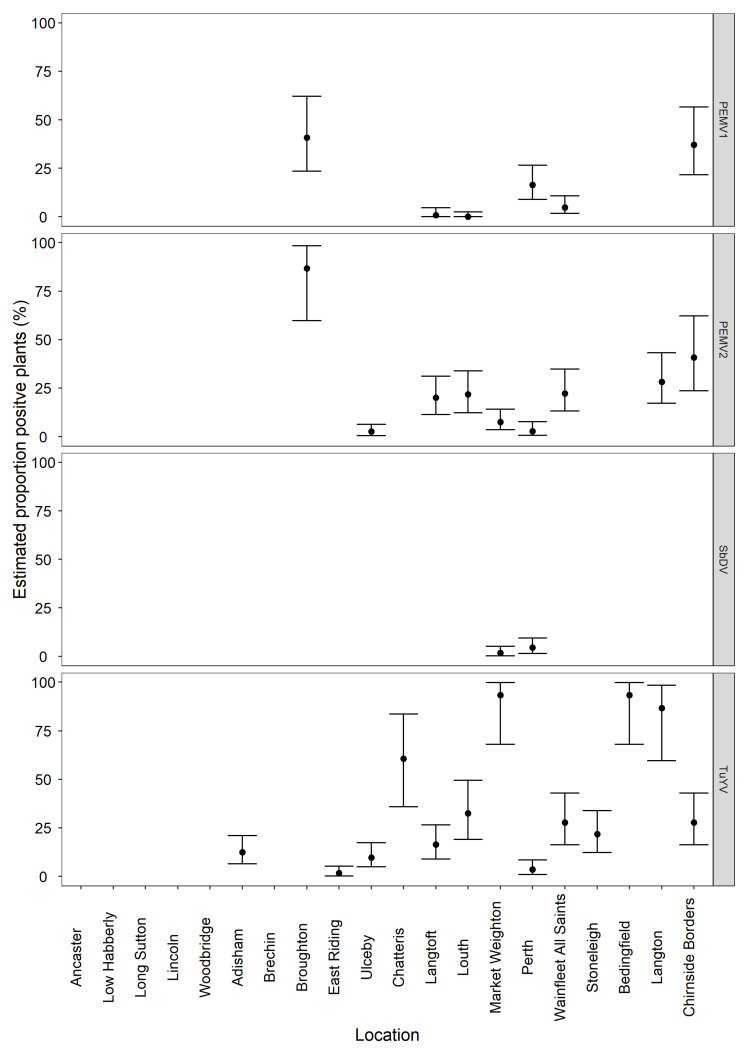
Graph showing the average incidence (%) with the upper and lower confidence values of pea enation mosaic virus-1 pea enation mosaic virus-2, soybean dwarf virus and turnip yellows virus at the 20 sites.

Samples from three of the whole crop pooled samples were re-sequenced at greater depth from library preparations previously produced for the initial runs. These were samples from sites Market Weighton, Perth and Chirnside. Sites Market Weighton and Perth were selected due to the presence of mixed infections including soybean dwarf virus partial sequences in the initial analysis, likely due to low concentration of the virus within the whole crop pooled sample possibly due to low incidence in the field (Figure 4). The sample from Chirnside was chosen due to high field incidence of multiple viruses. All three samples were late season and therefore thought more likely to be exposed to high vector pressure and potentially greater chance of virus infection which may have been previously missed in the initial sequencing run.

## 3. Results

### 3.1. Bulked Field Sample Inferred Viruses and Sub-Sample Real-Time RT-PCR Confirmation/Incidence

Of the twenty BFS samples tested, fourteen were identified as having at least one virus present. The viruses identified were turnip yellows virus (TuYV, Genus *Polerovirus*), pea enation mosaic virus-1 (PEMV-1, Genus; *Enamovirus*), pea enation mosaic virus-2 (PEMV-2, Genus; *Umbravirus*), pea enation mosaic virus-satellite RNA (PEMV satRNA) and soybean dwarf virus (SbDV, Genus *Luteovirus*). No virus was detected at six of the sites and no further testing was carried out on these samples. By HTS, TuYV was most commonly detected, being present at thirteen sites, PEMV-1 at four sites, PEMV-2 at nine sites, PEMV satRNA at three sites and SbDV at two sites. Results are presented in Figure 4, with more detail provided in Table S1.

From initial testing of the BFS by HTS seven genomes of TuYV, three genomes of PEMV-1 and seven genomes of PEMV-2 were obtained. Due to the nature of sampling and sequencing of isolates from pooled samples multiple viral contigs of each species were obtained, suggesting multiple isolates were present. Therefore, where a genome could be obtained a single representative isolate is presented, where a whole genome was not obtained, a partial sequence is presented. To give some indication of the amount of viral sequences present and to allow for a comparison across samples, reads per kilobase of transcript per million mapped reads (RPKM) values were calculated (Table S2). Representative genomes for the identified viruses TuYV, SbDV, PEMV-1, PEMV-2, PEMV satRNA PSbMV and BYMV were obtained from GenBank (accession numbers for all genomes are presented in Table S2). Due to the genetic variation present in TuYV, two representative genomes were used [36]. Each sample was mapped to each of the representative genomes using bwa-mem [37], and RPKM was calculated from the resulting bam file using BBTools’ pileup [38] command.

Where HTS indicated the presence of virus from a whole crop sample, real-time RT-PCR was used to both confirm infection and determine incidence of the virus from the corresponding mixed-bulk size samples. The calculated incidence of TuYV ranged from 2–93%, with seven sites having an incidence under 25% and six sites over 50%. PEMV-1 was present at about 40% incidence in two sites, and 16% and 5% incidence in the other two sites. The highest incidence of PEMV-2 found was 86%, five sites had an incidence between 20 and 40% and three had an incidence below 10%. SbDV was present at two sites with less than 5% incidence at both sites. Results are presented in Figure 4, with more detail provided in Table S1.

At sites Ulceby, Langtoft, Louth, Market Weighton and Langton HTS identified PEMV-2 but not PEMV-1. Due to the association of these two viruses, the sites were subjected to further testing by real-time RT-PCR. From further testing on four of these sites, only one bulked sample from Langtoft tested positive for PEMV-1. Results for Langtoft and Louth are shown in Figure 4 and Table S1.

As SbDV is a new record for the UK, the samples which tested positive by real-time RT-PCR were then tested by RT-PCR and submitted for Sanger sequencing (MWG GmbH). From Market Weighton one individual leaf sample and one bulked sample tested positive by both methods (GenBank accessions: OK492197, OK492196) and from Perth one individual leaf sample tested positive by both PCR methods (GenBank accession: OK492198).

### 3.2. Symptomatic Samples from Sites HTS Inferred Viruses and Real-Time RT-PCR Confirmation

Symptomatic samples from Market Weighton, Wainfleet, Eye and Langton were tested as bulks. HTS testing identified TuYV, PEMV-1, PEMV-2 and PEMV satellite RNA, Table 2. Either a whole genome or partial sequence representing each site has been uploaded to GenBank (Table S2).

The samples were then individually tested by real-time RT-PCR for TuYV, PEMV-1 and PEMV-2, Table 2. For each site, the real-time RT-PCR results were concordant with the HTS findings, the one exception being Langton sample 2, where a Ct of 35 was obtained for PEMV-2 but only TuYV was identified by HTS.

There was a difference in the identity of viruses indicated by HTS in the bulk field sample and the viruses detected in the symptomatic samples taken at that site. For example, at Market Weighton, SbDV was detected in the bulked field sample but not in the eight symptomatic samples.

### 3.3. General Surveillance Samples HTS Inferred Viruses and Real-Time RT-PCR Confirmation

In the nine general surveillance samples HTS inferred the presence of TuYV, PEMV-1, PEMV-2, PEMV satRNA, Pea seed-borne mosaic virus (PSbMV), Bean yellow mosaic virus (BYMV) and four contigs ranging from 200 to 400 nt of turnip yellows virus associated RNA (TuYVaRNA), Table 2. Either a whole genome or partial sequence representing each site has been uploaded to GenBank Table S2.

The samples were tested by real-time RT-PCR for TuYV, PEMV-1 and PEMV-2, three samples were also tested for PSbMV to confirm presence. Results of testing of the symptomatic samples by real-time RT-PCR in most cases were concordant with the viruses inferred by HTS. Exceptions to this are the Market Rasen sample, which tested positive for TuYV (Ct 24), PEMV-1 (Ct 32) and PEMV-2 (Ct 32). Further, both Ramsey-2 and Ramsey-4 tested positive for TuYV (Ct 14, Ct 14), PEMV-1 (Ct 30, Ct 32) and PEMV-2 (Ct 11, Ct 12), respectively.

### 3.4. HTS-Increased Depth Testing

As a quality control step to check whether any viruses had been missed on the initial testing, Bulked Field Samples (BFS) from three sites (sites Market Weighton, Perth and Chirnside) were re-analysed on the MiSeq at an increased sequencing depth. This increased depth equated to approximately a six-fold increase in depth of sequencing over the initial analysis. HTS inferred the presence of the same plant infecting viruses as on the initial run. Running the samples at greater depth did mean that more sequence data was obtained for each virus species at the three sites shown in Table S2. From initial testing, four contigs of SbDV of less than 550 nt were found at sites Market Weighton and Perth. By re-testing the samples at depth, a near-complete genome of SbDV was obtained from Market Weighton and a partial genome was obtained at Perth, representative sequences were uploaded to GenBank and accession numbers are presented in Table S2. Additionally, at Chirnside, 14 reads (forming contigs between 189 nt to 280 nt) with greater than 90% homology to Wuhan insect virus 21 (MN497793) were also obtained, an exemplar sequence has been uploaded to GenBank (GenBank accession: OK030797).

### 3.5. Historic Isolate

Testing by HTS inferred the presence of PSbMV, TuYV and SbDV in this sample, whole genomes were obtained for the three viruses, GenBank accession numbers and reads obtained by HTS can be found in Table S2. By initial screening this sample tested positive for PSbMV by ELISA. As a new record for the UK, the presence of SbDV was confirmed by real-time RT-PCR, RT-PCR, and Sanger sequencing (GenBank accession: OK492195).

## 4. Discussion

Crops can be host to a wide range of viruses, and with the movement of plants and changing climate it is important to determine a baseline for virus presence in a crop [39]. The aims of this study were to provide a baseline for pea viruses in the UK, to show the feasibility of integrating HTS into a survey programme using a BFS approach. This would enable screening a large area or crop for the presence of novel and unexpected viruses, and confirming presence, distribution and incidence in a single workflow. As there has been limited investigation into peas in the UK, this work provides updated information on the presence and incidence of viruses in UK pea crops.

In this study, six viruses, one satellite RNA and an associated RNA were detected by HTS across all 20 sites, and 28 symptomatic samples. This included viruses that were previously reported as present in UK peas; pea enation mosaic virus-1 (PEMV-1,Genus; *Enamovirus*), pea enation mosaic virus-2 (PEMV-2, Genus; *Umbravirus*), Pea seed-borne mosaic virus, bean yellow mosaic virus (PSbMV, BYMV, Genus; *Potyvirus*) and pea enation mosaic virus satellite RNA (PEMV satRNA). Two more viruses were also detected, turnip yellows virus (TuYV, Genus *Polerovirus*) and soybean dwarf virus (SbDV, Genus *Luteovirus*), and finally turnip yellows virus-associated RNA. TuYV represents a new host record for the UK, and the finding of SbDV is a new record for the UK.

TuYV is known to be present and widespread in the UK and is commonly found in oilseed rape (*Brassica napus* subsp *napus*), and has a wide host range including 13 plant families [40]. TuYV has been reported on peas and other legumes in Germany, Australia and New Zealand [41,42,43]. Across both the bulked field samples and symptomatic samples TuYV was the most common virus, present in 13 of the sites tested and ranging from 2–94% incidence. A survey of peas in New Zealand in 1993 found TuYV was widespread and occurred at a high incidence [42], similarly Gaafar et al. [17] found TuYV in at least half the sites tested over 3 years. An investigation into the incidence of TuYV in oilseed rape in three regions in England between 2007–2010 found TuYV in 26 of the 27 fields tested, with an incidence of 1–100%. While no recent studies have been done on TuYV incidence in England, the authors report that limited testing of commercial oilseed crops in 2018–2019 also found TuYV incidence between 0 and 100% [44].

Pea enation mosaic disease is characterised by the formation of enations (ridges of proliferated tissue) which form on the underside of leaves, but can also cause dwarfing, yellow mosaic and leaf curl [45,46,47]. Pre-1990 it was thought that pea enation mosaic disease was caused by one virus with a bipartite genome and a third non-essential RNA. However, characterisation of the 3 RNAs revealed that RNA1 (PEMV-1) and RNA2 (PEMV-2) are taxonomically different viruses which exist in a symbiotic association, with RNA3 (PEMV satRNA) which is not essential for infection and is reliant on PEMV-2 for replication [48,49,50]. Therefore, reports of pea enation mosaic from before 1990 are based on incidence of the disease, not the incidence of the viruses.

In this study HTS and subsequent testing detected PEMV-1 in only five of twenty sites and at incidence less than 40%. From Hagedorn’s work PEMV-1 may have been expected in more of the fields [1]. Aftab et al. [41] describe stunting and yellowing as typical symptoms of TuYV on peas, similar to those caused by PEMV-1. Where enations are not present, there could have be misidentification of viruses present, and symptoms previously attributed to PEMV-1 could also have been caused by TuYV. TuYV and PEMV-1 are both transmitted by aphids in a persistent manner, and there are aphid species which can transmit both viruses [40,51] so the aphids themselves are unlikely to lead to TuYV being found at a higher incidence than PEMV-1. TuYV has a wider host range, which could lead to more TuYV inoculum being present in the environment [40,47]. Gaafar et al. [17] sampled surrounding leguminous and non-leguminous plants, while they found TuYV in the non-leguminous plants, PEMV-1 and PEMV-2 were only seen in the peas. Many varieties of pea have genetic resistance to PEMV-1 which may also have an effect.

There were instances where PEMV-2 was identified without the PEMV-1 helper virus. As these two viruses are always thought to be present together, some samples from sites where only PEMV-2 was found were tested for PEMV-1, of these only one bulked sample tested positive. Further, in-depth HTS testing of Market Weighton did not identify PEMV-1 in the sample. Gaafar et al. [17] also found PEMV-2 by itself, and suggest that another virus is acting as the helper virus. Testing of individual plants in this study for incidence did not reveal plants infected with PEMV-2 to be commonly infected with another virus (aside from PEMV-1) (data not shown). Umbraviruses have been known to be spread by manual inoculation which could explain the occurrence of PEMV-2 without a helper virus, but further work is required to better understand the occurrence of this virus [52].

SbDV was detected at a low incidence at two sites and it was also found in the historic isolate. The historic isolate was sequenced as part of ongoing work to sequence isolates from the Fera Freeze-Dried Virus Collection. This pea isolate was originally diagnosed with PSbMV by ELISA. Testing by HTS inferred the presence of PSbMV, TuYV and SbDV. This virus has not been reported previously in the UK, but is known to be present in Germany [32] and Finland [53]. This shows the value in using HTS for surveillance as otherwise this virus would have been missed, and it shows the value of sequencing old isolates. Here the sequencing of a historic isolate strengthened the plant health context of the finding. Not only does this show that both TuYV and SbDV have been present in the UK for at least a decade it also shows the value of sequencing isolates for quality checking, as any symptoms present would have been attributed to PSbMV and would not have been investigated further.

PSbMV and BYMV were only observed in the single symptomatic samples. Both viruses are known to be present in the UK. PSbMV is a seed-transmitted virus, plants grown from infected seed become a source of inoculum, PSbMV can then be transmitted to surrounding plants by aphid vectors and wind-mediated contact transmission [54]. In the 1980s and early 1990s in the UK, work was done to screen seed for PSbMV to reduce incidence in crop, more recently seed screening and aphid control to prevent spread within the crop are used to control PSbMV (B Howard, 2020, personal communication). Presence of BYMV was not confirmed as this virus has been known to be present on UK peas since 1940, recorded as pea mosaic virus, and only occurred in one sample [1,45].

Similar to Gaafar et al. [17] TuYVaRNA was only found in two samples but not at sites with high TuYV incidence. Both satellite viruses and associated RNA can have an effect on symptom severity, associated RNAs have been known to increase symptom severity whereas PEMV satRNA has been shown to attenuate symptoms on *Nicotiana benthamiana* but no effect was seen on peas [50,55]. The use of HTS allows findings of novel, satellites and unexpected viruses or sub-viral agents and confirmation and characterization of every finding can be time-consuming, difficult and costly [8]. As pea enation mosaic disease is known to be present in the UK, the finding of PEMV satRNA is expected, while TuYVaRNA and Wuhan insect virus 21 have not been previously identified in UK. Both TuYVaRNA and Wuhan insect virus 21 have been reported from Germany [17,43].

Overall, the results from the real-time RT-PCR testing correlated with the viruses inferred from HTS data with a few minor discrepancies. These findings could be explained by sensitivity or sub-sampling strategy. Viruses of the *Solemoviridae* (formerly Luteoviridae) are phloem-limited [56], if little phloem material was taken during extraction, this could also explain why HTS did not detect TuYV.

In some cases viruses were not seen in both the whole crop sample and the symptomatic field samples, there was no trend of more viruses being seen in the symptomatic plants rather than the BFS. Gaafar et al. [17] tested both symptomatic and asymptomatic samples from each site separately, and found more viruses in the symptomatic samples. For our study, our samples were taken randomly according to a set sample pattern regardless of symptom status. The likely presence of infected but asymptomatic plants and random sampling could explain why the BFS results do not differ greatly from the results of the symptomatic plants.

BFS were initially run as single libraries by HTS. Three of the twenty sites were then chosen for in-depth sequencing, in which they were the only samples on the run. All three sites were chosen because they were later in the season and had a greater chance of virus infection, additionally short contigs of SbDV were found in the Market Weighton and Perth sites. In all three cases, the same plant viruses were detected by both the initial HTS and the in-depth run, suggesting that running the BFS as a single library is sufficient to detect the presence of viruses. In all three cases running in-depth increased the depth and the genome coverage of the virus sequences recovered (Table S2). After the in-depth testing, a near complete genome of SbDV was obtained from Market Weighton and a partial genome was obtained from Perth. Interestingly, Perth had a higher incidence of SbDV than Market Weighton but in both HTS runs more SbDV nucleotides were obtained from Market Weighton. This suggests that the chance of detecting virus in the sample is not only affected by its incidence in the field, but also its titre in the sample.

As the study was to focus on viruses, an rRNA depleted total RNA HTS method was chosen because the majority of plant viruses have an RNA genome or intermediary RNA step, it has also been previously shown to be able to detect viruses with either an RNA or DNA genome [17,57].

Testing the BFS by HTS identified prominent viruses at the site, which could then be confirmed using real-time RT-PCR. This process reduced further testing, as samples were only tested for viruses indicated to be present. Whereas in a traditional sample, every sample or multiple small bulks would have to be tested for the full suite of viruses selected for the study.

For the incidence testing, mixed bulk sample sizes were chosen to find a balance between cost and confidence intervals for analysing both high and low virus incidence. Table S3 gives estimates of prevalence with 95% confidence intervals for 15 7-plant pools and 15 1-plant pools. Table S4 gives estimates where the same number of plants are tested using the same number tests in 30 4-plant pools. The main difference between the two approaches is finer quantitative resolution of central estimates and smaller confidence intervals for high prevalence estimates where two pool sizes are used (Figure S1).

This study describes a proof of concept of a generic approach for surveillance, where HTS is integrated into a crop-specific field survey. Prior to using this method on other crop types, there are some critical points to consider. For this study an RNA-focused approach was used because the majority of plant viruses have an RNA genome or RNA step, and the method used can detect DNA viruses. The type of crop could also have an effect on the survey, a 100 m^2^ area can be used for monoculture field crops but wouldn’t be as useful when mixed species are present or for sampling trees which take up a lot more space. Pea leaves are very easy to extract and a high yield of RNA was recovered after extraction, but if the material has inhibitors present, such as phenolics or polysaccharides, this could make the extraction process more difficult. Peas have a very small window for testing prior to being harvested which puts pressure on a fast turn-around for results and there is no option for re-testing, this also means the results obtained are true for that point in time. Where plants spend longer in the ground, e.g., trees, it would be possible to re-sample from the same site at different times points or sample different areas. In this study, the aim was to identify which viruses are present in UK peas to inform a baseline so re-testing was not necessary. Another limitation of this method is that it could not be used to demonstrate that an area is free from a particular virus as, if the virus is present at a very low incidence it could be missed by this testing. For further testing a whole genome was not required, as with the confirmation of SbDV, whereas if the aim of a study was to investigate genetic variability, testing the samples as BFS would not be recommended due to the relative limited coverage of individual genomes as demonstrated by the relative differences in depth of genome coverage between BFS, smaller bulks and individual samples (See Table S2).

Prior to this study TuYV had not been recorded from UK peas. This study also provides the first record of SbDV in the UK. A conventional survey would have included testing smaller bulks for viruses known to occur in the UK (e.g., PSbMV) and pea necrotic yellow dwarf virus (PNYDV) a virus which has spread through pea crops in Europe. PSbMV was only found in three symptomatic samples and PNYDV was not found in this initial work. Sequencing of historic isolates can also give greater understanding of the plant health impact of more recent virus discoveries [58]. The finding of TuYV and SbDV in a historical isolate shows that these viruses have been present in the UK for at least a decade. Further work is needed to investigate the symptoms, yield loss, key aphid vectors and sources of both TuYV and SbDV in the UK.

This work provides a comprehensive snapshot of the incidence and prevalence of viruses in UK pea crops. The virus baseline being established here can help inform effective crop management in the future. Within this study twenty BFS, each comprising of 120 leaves, were tested by HTS allowing for identification of unexpected viruses. Despite pooling a large number of leaves into a single BFS, viruses present at a low incidence were still detected by HTS. Three of the BFS were re-tested in-depth by HTS and no further plant viruses were identified, suggesting that the initial run was sufficient for the intended purpose of identifying candidate viruses for subsequent confirmation testing. By using HTS to identify candidate viruses, the testing of smaller mixed-size bulks could be focused for viruses inferred from sequencing, thus reducing confirmation testing and consequently incurred costs by comparison to running a similar exercise using targeted diagnostic approaches. The added advantage of such an approach is that the initial screening method is generic and could therefore be applied to other crops. Whilst the method here is applied as a general survey of pea crops, this testing could be used within routine surveys e.g., certification or in regulatory systems. For application in other crops considerations would need to be given to the specific traits of that crop (e.g., leaf structure, cropping pattern) and those of the target pathogens (e.g., within plant distribution, homogenous or aggregated field distribution) in the scope of the study. The value of a generic workflow approach such as this is that it can give information on the presence, distribution and incidence of viruses in a single sampling exercise. Within regulatory testing the sample size is governed by the detection threshold of a given pathogen and the efficacy of the test method being applied, as recommended in International Standards of Phytosanitary management (ISPM) standard 31 [59]. Here 120 leaf samples were taken as a proof of principle, this equates to approximately a 95% confidence of detecting a 3% pathogen presence (assuming a 100% efficacy of the test method). However, if the approach were applied specifically for the detection of low incidence pathogens, such as recent incursions of a regulated pathogen, a greater number of samples would need to taken from each site. However, this challenge is not specific to this workflow and applies equally to any method being used in surveillance.

The reliability of this testing approach at the limit of detection is currently unknown, but within this study there are instances of virus being detected by HTS at less than 4% calculated incidence at the site. Three of the Bulked Field Samples were re-tested by HTS to give a greater depth of sequencing and no additional viruses were found suggesting the initial comparatively low sequence depth was adequate. If using this approach in routine surveillance, re-running a sub-set of BFS could be used as a quality assurance measure, however this would be limited by cost implications. The workflow described here is cost-effective as, by comparison to traditional targeted approaches, only candidate viruses identified by HTS are the focus of confirmation testing. Whereas, in a traditional survey samples would be tested by a pre-determined suite of specific tests for known viruses and/or emerging threats. While, this could give evidence that the surveyed area is free from a particular virus, it would not give the added benefit of detecting viruses which would not be included in a fixed suite of targeted tests, such as the TuYV and SbDV in this study.

## Figures and Tables

**Table 1:** Real-time RT-PCR and RT-PCR assays used within this work.

Primer Set		Sequence (5′–3′)	Source
Real-time RT-PCR			
Pea enation mosaic virus-1	PEMV-1-4838 F	YCT AAT ACC TAG TAG CAA AGG GAA GTT T	This study
	PEMV-1-4815	CCC CAA CAT CCA TCA GCT TTT C	
	PEMV-1-4891 Pe1	FAM-TCC AAG CAG TGA AGA GCA TTG GYG-TAMRA	
	PEMV-1-4891 Pe2	FAM-TCC AAG CAG TGA AGA GTA TTG GAG-TAMRA	
Pea enation mosaic virus-2	PEMV-2-1347 F	CTG AAA AGA TAA AYT TCA CAG CCA AA	This study
	PEMV-2-1395 R	AAT CGT GGA TCC CTA GGC TGT A	
	PEMV-2-1375 Pe	FAM-TGA CCC CGC CCC TCG TGT G-TAMRA	
Pea seed-borne mosaic virus	PSbMV-9129 F1	TGA CAT HTC AAA CAC TCG AGC AA	This study
	PSbMV-9129 F2	TGA CAT HTC AAA CAC TCG CGC AA	
	PSbMV-9190 R1	TGT CWC CAA YCC CRT ACT CTT G	
	PSbMV-9155 Pe1	FAM-AGA GCC AGT TYG ATA AYT GGT GGA GGG-TAMRA	
	PSbMV-9155 Pe2	FAM-AGA GCC AAT TYG ATA AYT GGT GGA GAG-TAMRA	
Soybean dwarf virus	SbDV-F	TGG CTA TTA TAG AAT GGT GCG TAA AC	[30]
	SbDV-R	GCC ATG GAA ATG AGG GAA TG	
	SbDV-Pe	FAM-AGC ATA TCC AAA GAC GC-MGB	
Turnip yellows virus	TuYV-F2	GCC GCT TGT TTC TCA GTT CTG	[31]
	TuYV-R2	GAC TAA CCA CGA GTA AAG AAG CTC AA	
	TuYV-P2	FAM-ACG AGT TGC GGC ACG ATC CAG C-TAMRA	
RT-PCR			
Soybean dwarf virus	SbDV-F	GTC TAC CTA AAA ATT TCA AAG AAT CTG	[32]
	SbDV-R	CGG ACC CGG TTC TCC GTC TA	

**Table 2:** Results for testing of symptomatic samples. The nine general surveillance samples from Eastern England were tested by HTS individually, the symptomatic samples from sites were tested as bulks representing the site. Table also shows confirmation of HTS findings by real-time RT-PCR. Samples which were not tested listed as nt.

Site	Sub- Sample	HTS Result	Results of Real-Time RT-PCR Confirmation			
			TuYV	PEMV-1	PEMV-2	PSbMV
Market Rasen		Fragment of turnip yellows virus associated RNA (TuYVaRNA)	Positive	Positive	Positive	Nt
Ramsey-1		TuYV, PEMV-1, PEMV-2	Positive	Positive	Positive	Nt
Ramsey-2		TuYV, PEMV-2	Positive	Positive	Positive	Nt
March		TuYV, PEMV-1, PEMV-2, Pea seed-borne mosaic virus (PSbMV), Fragments of TuYVaRNA	Positive	Positive	Positive	Positive
Ramsey-3		TuYV, PEMV-1, PEMV-2, PSbMV	Positive	Positive	Positive	Positive
Ramsey-4		TuYV, PEMV-2	Positive	Positive	Positive	Nt
The Deepings		TuYV, PEMV-1, PEMV-2, PEMV satRNA, bean yellow mosaic virus (BYMV)	Positive	Positive	Positive	Nt
Cambridge-1		TuYV, PEMV-1, PEMV-2, PSbMV	Positive	Positive	Positive	Positive
Cambridge-2		PEMV-1, PEMV-2, PEMV satRNA	Negative	Positive	Positive	Nt
Market Weighton	1	TuYV, PEMV-1, PEMV-2	Positive	Positive	Positive	Nt
Market Weighton	2		Positive	Negative	Negative	Nt
Market Weighton	3		Positive	Positive	Positive	Nt
Market Weighton	4		Positive	Negative	Positive	Nt
Market Weighton	5		Positive	Negative	Positive	Nt
Market Weighton	6		Positive	Negative	Negative	Nt
Market Weighton	7		Positive	Positive	Negative	Nt
Market Weighton	8		Positive	Negative	Negative	Nt
Wainfleet	1	TuYV, PEMV-1, PEMV-2, PEMV satRNA	Positive	Negative	Positive	Nt
Wainfleet	2		Positive	Positive	Positive	Nt
Wainfleet	3		Positive	Positive	Positive	Nt
Wainfleet	4		Positive	Positive	Positive	Nt
Wainfleet	5		Positive	Positive	Positive	Nt
Wainfleet	6		Positive	Positive	Positive	Nt
Eye	1	TuYV, PEMV-2	Positive	Negative	Negative	Nt
Eye	2		Positive	Negative	Negative	Nt
Eye	3		Positive	Negative	Positive	Nt
Langton	1	TuYV	Positive	Negative	Negative	Nt
Langton	2		Positive	Negative	Positive	Nt

## Data Availability

All sequences have been deposited in GenBank, accession numbers can be found in Table S2. Additional sequences deposited under numbers: OK030797, OK492195–OK492198.

## Supplementary Materials

The following are available online at https://www.mdpi.com/article/10.3390/v13122530/s1, Figure S1: Central estimates and 95 confidence intervals for two different sizes of pools used to test 120 plants. Table S1: HTS and incidence results for site testing. Table S2. Table shows the reads per kilobase of transcript per million mapped reads (RKPM) for each sample tested by HTS SM. Table S3: Central estimates and 95 confidence intervals for prevalence estimates based on testing 15 7-plant pools and 15 1-plant pools. Table S4: Central estimates and 95 confidence intervals for prevalence estimates based on testing 30 4-plant pools.
