# Supplementary material for: Integrating High throughput Sequencing into Survey Design Reveals Turnip Yellows Virus and Soybean Dwarf Virus in Pea (Pisum Sativum) in the United Kingdom
Source: Viruses. 2021 Dec 16;13(12):2530. doi: 10.3390/v13122530 (PMC8707713; doi:10.3390/v13122530)

Supplementary Figure S1: Central estimates and 95% confidence intervals for two different sizes of pools used to test 120 plants

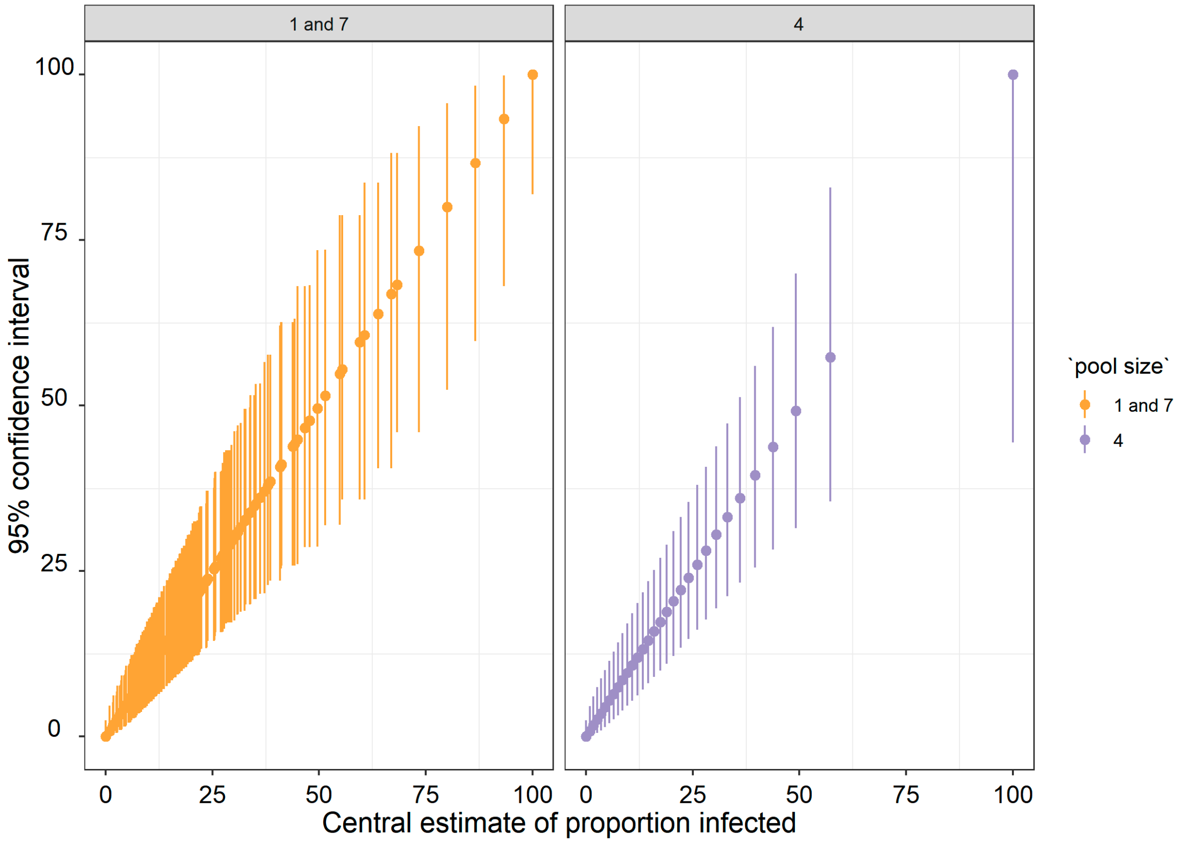

Supplement: Supplementary file 1 [file viruses-13-02530-s001.zip › Supplementary Figure S1 Central estimates and 95 confidence intervals for two different sizes of pools used to test 120 plants.pdf]
