# Supplementary material for: Integrating High throughput Sequencing into Survey Design Reveals Turnip Yellows Virus and Soybean Dwarf Virus in Pea (Pisum Sativum) in the United Kingdom
Source: Viruses. 2021 Dec 16;13(12):2530. doi: 10.3390/v13122530 (PMC8707713; doi:10.3390/v13122530)
Supplement: Supplementary file 1 [file viruses-13-02530-s001.zip › Supplementary Table S1 HTS and incidence results for site testing.pdf]

Supplementary Table S1. HTS and incidence results for site testing. Table shows the site name, sampling date, initial result from high-throughput sequencing (HTS) and the results from real-time RT-PCR testing. The incidence of virus is given as a percentage, alongside the upper and lower incidence values. Where no result is shown, samples were not tested.

| | | | | TuYV virus estimate (%) | | | PEMV-1 virus estimate (%) | | | PEMV-2 virus estimate (%) | | | SbDV virus estimate (%) | | |
|---|---|---|---|---|---|---|---|---|---|---|---|---|---|---|---|
| Site | Location | Date of sampling | HTS result | Result Estimate | Lower | Upper | Result Estimate | Lower | Upper | Result Estimate | Lower | Upper | Result Estimate | Lower | Upper |
| 1 | Ancaster | 30.04.2019 | No virus detected | | | | | | | | | | | | |
| 2 | Kidderminster | 13.05.2019 | No virus detected | | | | | | | | | | | | |
| 3 | Long Sutton | 14.05.2019 | No virus detected | | | | | | | | | | | | |
| 4 | Lincoln | 20.05.2019 | No virus detected | | | | | | | | | | | | |
| 5 | Woodbridge | 22.05.2019 | No virus detected | | | | | | | | | | | | |
| 6 | Canterbury | 23.05.2019 | TuYV | 12.46 | 6.38 | 21.05 | | | | | | | | | |
| 7 | Finavon | 28.05.2019 | No virus detected | | | | | | | | | | | | |
| 8 | Salisbury | 28.05.2019 | PEMV-1 PEMV-2 | | | | 40.8 | 23.55 | 62.15 | 86.67 | 59.69 | 98.34 | | | |
| 9 | East Riding | 29.05.2019 | TuYV | 1.71 | 0.21 | 5.16 | | | | | | | | | |
| 10 | Ulceby | 10.06.2019 | TuYV PEMV-2 | 9.71 | 4.97 | 17.38 | | | | 2.57 | 0.54 | 6.25 | | | |
| 11 | Chatteris | 18.06.2019 | TuYV | 60.62 | 35.9 | 83.67 | | | | | | | | | |
| 12 | Langtoft | 20.06.2019 | TuYV, PEMV-2 | 16.39 | 8.93 | 26.56 | 0.85 | 0.02 | 4.66 | 20.05 | 11.31 | 31.14 | | | |
| 13 | Louth | 26.06.2019 | TuYV, PEMV-2 | 32.56 | 19.03 | 49.52 | 0 | 0 | 2.47 | 21.8 | 12.32 | 33.83 | | | |
| 14 | Market Weighton | 03.12.2019 | TuYV, PEMV-2, SbDV | 93.33 | 68.08 | 99.83 | | | | 7.49 | 3.49 | 14.07 | 1.71 | 0.21 | 5.16 |
| 15 | Perth | 03.07.2019 | TuYV, PEMV-1, PEMV-2, SbDV | 3.62 | 1.01 | 8.43 | 16.39 | 8.93 | 26.56 | 2.71 | 0.63 | 7.67 | 4.53 | 1.51 | 9.41 |
| 16 | Wainfleet | 10.07.2019 | TuYV, PEMV-1, PEMV-2, PEMV-satRNA | 27.77 | 16.27 | 42.99 | 4.81 | 1.74 | 10.73 | 22.23 | 13.25 | 34.74 | | | |
| 17 | Stoneleigh | 16.07.2019 | TuYV | 21.8 | 12.32 | 33.83 | | | | | | | | | |

| | | | | | | | | | | | | | | | |
|---|---|---|---|---|---|---|---|---|---|---|---|---|---|---|---|
| 18 | Eye | 17.07.2019 | TuYV, PEMV-satRNA | 93.33 | 68.08 | 99.83 | | | | | | | | | |
| 19 | Langton | 18.07.2019 | TuYV, PEMV-2 | 86.67 | 59.69 | 98.34 | | | | 28.2 | 17.09 | 43.25 | | | |
| 20 | Chirnside | 24.07.2019 | TuYV, PEMV-1, PEMV-2, PEMV-satRNA | 27.77 | 16.27 | 42.99 | 37.15 | 21.63 | 56.58 | 40.8 | 23.55 | 62.15 | | | |
