# Supplementary material for: Integrating High throughput Sequencing into Survey Design Reveals Turnip Yellows Virus and Soybean Dwarf Virus in Pea (Pisum Sativum) in the United Kingdom
Source: Viruses. 2021 Dec 16;13(12):2530. doi: 10.3390/v13122530 (PMC8707713; doi:10.3390/v13122530)
Supplement: Supplementary file 1 [file viruses-13-02530-s001.zip › Supplementary Table S2. Table shows the reads per kilobase of transcript per million mapped reads (RKPM) for each sample tested by HTS SM.pdf]

Supplementary Table S2. Table shows the reads per kilobase of transcript per million mapped reads (RPKM) for each sample tested by HTS, Equation RPKM = $(10^9 \times C)/(N \times L)$, where C is the number of unique high-quality reads that mapped to the transcript, N is the total number of unique high-quality reads in the library, and L is the length of the transcript. The table also gives the GenBank accession number for the representative sequence. Sample type- BFS- bulk field sample, ID-BFS- in depth bulk field sample, BS- bulked symptomatic samples, IS- individual symptomatic sample, and HI- historic isolate.

Sample type	Virus	TuYV	TuYV	SbDV	PEMV-2	PEMV-1	PSbMV	BYMV	PEMV Sat	GenBank Accession Numbers for sequences obtained in this study
	Accession	NC_003743.1	MN497802.1	NC_003056.1	NC_003853.1	NC_003629.1	NC_001671.1	NC_003492.1	U03564.1	
	Length	5641	5577	5853	4253	5706	9924	9532	717	
BFS	Ancaster	1.0	1.0	0.0	0.0	0.0	0.0	0.0	0.0	
BFS	Kidderminster	0.0	0.0	0.0	0.0	0.0	0.0	0.0	0.0	
BFS	Long Sutton	0.0	0.0	0.0	0.0	0.0	0.0	0.0	0.0	
BFS	Lincoln	0.0	0.0	0.0	0.0	0.0	0.0	0.0	0.0	
BFS	Woodbridge	0.0	0.0	0.0	0.0	0.1	0.0	0.0	0.0	
BFS	Canterbury	5.4	36.7	0.0	0.0	0.5	1.0	0.2	0.0	TuYV: OK030767
BFS	Finavon	0.0	0.0	0.0	0.0	0.0	0.0	0.0	0.0	
BFS	Salisbury	0.0	0.2	0.0	776.4	2593.6	0.1	0.1	0.0	PEMV-1: OK030715<br>PEMV-2: OK030729
BFS	East Riding	0.9	2.1	0.0	0.0	0.0	0.0	0.0	0.0	TuYV: OK030768
BFS	Ulceby	5.7	21.9	0.0	11.0	0.6	0.0	0.0	10.0	PEMV-2: OK030758<br>TuYV: OK030769

BFS	Chatteris	58.1	242.6	0.2	0.0	0.7	0.3	0.4	0.0	TuYV: OK030770
BFS	Langtoft	6.4	33.9	0.4	311.9	0.8	0.7	0.7	0.0	PEMV-2: OK030730<br>TuYV: OK030771
BFS	Louth	26.6	93.5	0.0	557.0	0.0	0.1	0.5	0.0	PEMV-2: OK030731<br>TuYV: OK030772
BFS	Market Weighton	56.5	287.2	2.2	23.5	0.0	0.1	0.1	0.0	PEMV-2: OK030732<br>SbDV: OK030748<br>TuYV: OK030773
ID-BFS	Market Weighton	58.4	280.9	3.4	22.5	0.2	0.2	0.3	0.0	PEMV-2: OK030795<br>SbDV: OK030799<br>TuYV: OK030750
BS	Market Weighton	78.6	226.7	0.1	11.6	76.8	0.7	0.8	0.0	PEMV-1: OK030716<br>PEMV-2: OK030796<br>TuYV: OK030774
BFS	Perth	0.2	1.2	0.8	64.8	694.6	0.7	1.2	0.0	PEMV-1: OK030717<br>PEMV-2: OK030735<br>SbDV: OK030749<br>TuYV: OK030775
ID-BFS	Perth	0.1	1.2	1.2	63.9	735.2	0.6	1.0	0.0	PEMV-1: OK030718

										PEMV-2: OK030733<br>SbDV: OK030751<br>TuYV: OK030776
BFS	Wainfleet	9.1	43.1	0.0	425.0	10.7	0.0	0.0	444.1	PEMV-1: OK030766<br>PEMV-2: OK030734<br>PEMV-satRNA: OK030761<br>TuYV: OK030777
BS	Wainfleet	172.0	593.3	0.1	5422.6	8825.0	0.1	0.2	15152.3	PEMV-1: OK030719<br>PEMV-2: OK030736<br>PEMV-satRNA: OK030762<br>TuYV: OK030778
BFS	Stoneleigh	2.5	8.8	0.0	0.0	0.1	0.1	0.0	0.0	TuYV: OK030779
BFS	Eye	20.8	48.9	0.4	0.8	0.2	0.3	0.9	96.8	PEMV-satRNA: OK030763<br>TuYV: OK030780
BS	Eye	100.9	802.5	0.0	209.9	0.5	0.1	0.2	0.0	PEMV-2: OK030737<br>TuYV: OK030781
BFS	Langton	55.6	273.3	0.2	140.1	0.4	0.1	0.3	0.0	PEMV-2: OK030740

										TuYV: OK030782
BS	Langton	130.5	533.9	0.4	0.1	0.7	0.3	0.2	0.0	TuYV: OK030783
BFS	Chirnside	2.4	9.2	0.0	235.5	470.1	0.5	0.1	26.5	PEMV-1: OK030720<br>PEMV-2: OK030738<br>PEMV-satRNA: OK030760<br>TuYV: OK030784
ID-BFS	Chirnside	3.1	9.2	0.1	247.0	503.0	0.4	0.4	24.7	PEMV-1: OK030727<br>PEMV-2: OK030739<br>PEMV-satRNA: OK030759<br>TuYV: OK030785
IS	Market Rasen	0.6	0.5	0.0	0.0	0.6	0.0	0.0	0.0	TuYVaRNA: OK030786
IS	Ramsey-1	2899.4	7769.4	0.2	26732.8	59323.1	0.0	0.0	0.0	PEMV-1: OK030721<br>PEMV-2: OK030741<br>TuYV: OK030798
IS	Ramsey-2	1794.7	7734.7	0.0	7533.2	0.7	0.0	0.4	0.0	PEMV-2: OK030742<br>TuYV: OK030788
IS	March	1659.2	4188.1	0.1	9861.7	15667.7	155.9	1.9	0.0	PEMV-1: OK030722

										PEMV-2: OK030743<br>PSbMV: OK030753<br>TuYV: OK030789<br>TuYVaRNA: OK030787
IS	Ramsey-3	67.0	600.9	0.1	19040.5	39555.5	7.5	0.3	0.0	PEMV-1: OK030723<br>PEMV-2: OK030744<br>PSbMV: OK030754<br>TuYV: OK030790
IS	Ramsey-4	6144.2	11323.7	0.0	3703.6	0.2	0.2	0.1	0.0	PEMV-2: OK030728<br>TuYV: OK030791
IS	The Deepings	6454.4	15091.5	0.0	29754.0	38626.4	0.2	35.8	53.8	PEMV-1: OK030724<br>PEMV-2: OK030745<br>PEMV-satRNA: OK030764<br>BYMV: OK030755<br>TuYV: OK030792
IS	Cambridge-1	3026.1	14705.9	0.5	12769.7	84.3	271.5	2.2	76062.0	PEMV-1: OK030725<br>PEMV-2: OK030746<br>PSbMV: OK030756

										TuYV: OK030793
IS	Cambridge-2	0.2	0.2	0.0	28709.2	53909.8	0.0	0.1	25462.8	PEMV-1: OK030726<br>PEMV-2: OK030747<br>PEMV-satRNA: OK030765
HI	East Anglia	9920.7	16824.6	2752.9	7529.3	0.2	1595.3	0.5	0.0	PSbMV: OK030757<br>SbDV: OK030752<br>TuYV: OK030794
