# Supplementary material for: Integrating High throughput Sequencing into Survey Design Reveals Turnip Yellows Virus and Soybean Dwarf Virus in Pea (Pisum Sativum) in the United Kingdom
Source: Viruses. 2021 Dec 16;13(12):2530. doi: 10.3390/v13122530 (PMC8707713; doi:10.3390/v13122530)
Supplement: Supplementary file 1 [file viruses-13-02530-s001.zip › Supplementary Table S3 Central estimates and 95 confidence intervals for prevalence estimates based on testing 15 7-plant pools and 15 1-plant pools.pdf]

Supplementary *Table S3: Central estimates and 95% confidence intervals for prevalence estimates based on testing 15 7-plant pools and 15 1-plant pools*

7-plant pools +ve	1-plant pools +ve	Prevalence (%) Estimate	95% confidence interval	
0	0	0	0	2.47
0	1	0.83	0.02	3.37
0	2	1.67	0.21	4.69
0	3	2.5	0.54	6.18
0	4	3.33	0.98	7.67
1	0	0.85	0.02	4.66
1	1	1.71	0.21	5.16
1	2	2.57	0.54	6.25
1	3	3.42	0.98	7.68
1	4	4.28	1.5	9.18
2	0	1.76	0.24	6.17
2	1	2.64	0.55	6.81
2	2	3.52	0.98	7.81
2	3	4.4	1.5	9.2
2	4	5.28	2.1	10.73
2	5	6.17	2.76	12.35
3	0	2.71	0.63	7.67
3	1	3.62	1.01	8.43
3	2	4.53	1.51	9.41
3	3	5.44	2.1	10.78
3	4	6.35	2.76	12.36
3	5	7.27	3.49	14.05
4	0	3.72	1.14	9.18
4	1	4.66	1.55	10.07
4	2	5.6	2.11	11.06
4	3	6.55	2.76	12.43
4	4	7.49	3.49	14.07
4	5	8.44	4.04	15.39
4	6	9.4	4.61	16.61
5	0	4.81	1.74	10.73
5	1	5.78	2.19	11.76
5	2	6.76	2.79	12.8
5	3	7.74	3.5	14.18
5	4	8.72	4.3	15.89
5	5	9.71	4.97	17.38
5	6	10.7	5.33	18.09
6	0	5.97	2.43	12.35
6	1	6.98	2.91	13.53
6	2	8	3.55	14.64
6	3	9.02	4.31	16.06
6	4	10.05	4.97	17.43
6	5	11.09	5.61	18.75
6	6	12.13	6.38	20.3
6	7	13.18	7.18	21.98
7	0	7.22	3.2	14.05
7	1	8.28	3.71	15.39
7	2	9.35	4.39	16.61
7	3	10.42	5.21	18.08
7	4	11.5	5.99	19.6

7	5	12.59	6.72	21.08
7	6	13.69	7.55	22.75
7	7	14.8	7.99	23.66
8	0	8.59	4.05	15.86
8	1	9.7	4.61	17.37
8	2	10.83	5.33	18.73
8	3	11.96	6.2	20.3
8	4	13.1	7.1	21.98
8	5	14.26	7.56	22.81
8	6	15.43	8.46	24.64
8	7	16.6	9.39	26.56
8	8	17.79	9.91	27.65
9	0	10.09	5	17.84
9	1	11.27	5.62	19.52
9	2	12.46	6.38	21.05
9	3	13.67	7.31	22.75
9	4	14.89	7.99	23.92
9	5	16.12	8.88	25.61
9	6	17.38	9.89	27.65
9	7	18.64	10.49	28.84
9	8	19.93	11.31	30.51
9	9	21.23	12.28	32.52
10	0	11.76	6.03	19.96
10	1	13.02	6.74	21.85
10	2	14.29	7.58	23.66
10	3	15.59	8.46	25.29
10	4	16.91	9.39	26.94
10	5	18.25	10.33	28.66
10	6	19.61	11.27	30.51
10	7	20.99	12.27	32.52
10	8	22.4	13.33	34.75
10	9	23.83	14.5	37.23
11	0	13.64	7.18	22.29
11	1	15	8.09	24.63
11	2	16.39	8.93	26.56
11	3	17.8	9.91	28.49
11	4	19.25	10.98	30.44
11	5	20.72	12.1	32.5
11	6	22.23	13.25	34.74
11	7	23.77	14.46	37.23
11	8	25.34	14.5	37.25
11	9	26.95	15.79	40.04
11	10	28.6	17.23	43.25
12	0	15.8	8.77	25.51
12	1	17.3	9.57	27.62
12	2	18.84	10.49	29.9
12	3	20.41	11.56	32.22
12	4	22.03	12.79	34.63
12	5	23.69	14.13	37.19
12	6	25.41	14.5	37.64
12	7	27.17	15.79	40.2
12	8	28.99	17.23	43.31
12	9	30.88	18.46	46.18
12	10	32.82	19.94	49.53
12	11	34.83	20.81	51.57
13	0	18.36	10.41	28.83
13	1	20.05	11.31	31.14

13	2	21.8	12.32	33.83
13	3	23.61	13.51	36.73
13	4	25.49	14.61	39.07
13	5	27.44	15.84	41.4
13	6	29.48	17.25	44.08
13	7	31.6	18.86	47.4
13	8	33.83	19.94	49.73
13	9	36.16	21.6	53.34
13	10	38.6	23.54	57.63
13	11	41.16	25.86	62.62
13	12	43.85	25.86	62.62
13	13	46.65	28.63	68.04
14	0	21.52	12.28	32.64
14	1	23.5	13.34	35.3
14	2	25.58	15.19	39.99
14	3	27.77	16.27	42.99
14	4	30.09	17.53	46.16
14	5	32.56	19.03	49.52
14	6	35.18	20.81	53.27
14	7	38	22.9	57.61
14	8	41.02	25.36	62.62
14	9	44.26	25.86	63.16
14	10	47.74	28.63	68.23
14	11	51.46	31.93	73.55
14	12	55.41	35.86	78.76
14	13	59.56	35.89	78.77
14	14	63.87	40.54	83.67
14	15	68.28	46.02	88.18
15	0	25.7	15.76	40.03
15	1	28.2	17.09	43.25
15	2	30.91	18.47	47.02
15	3	33.88	20.01	51.57
15	4	37.15	21.63	56.58
15	5	40.8	23.55	62.15
15	6	44.91	26.02	68.04
15	7	49.57	28.72	73.5
15	8	54.81	31.98	78.75
15	9	60.62	35.9	83.67
15	10	66.87	40.54	88.18
15	11	73.38	46.02	92.21
15	12	80.01	52.38	95.67
15	13	86.67	59.69	98.34
15	14	93.33	68.08	99.83
15	15	100	81.9	100
