# Supplementary material for: Integrating High throughput Sequencing into Survey Design Reveals Turnip Yellows Virus and Soybean Dwarf Virus in Pea (Pisum Sativum) in the United Kingdom
Source: Viruses. 2021 Dec 16;13(12):2530. doi: 10.3390/v13122530 (PMC8707713; doi:10.3390/v13122530)
Supplement: Supplementary file 1 [file viruses-13-02530-s001.zip › Supplementary Table S4 Central estimates and 95 confidence intervals for prevalence estimates based on testing 30 4-plant pools.pdf]

Supplementary *Table S4: Central estimates and 95% confidence intervals for prevalence estimates based on testing 30 4-plant pools*

4-plant pools +ve	Prevalence (%) Estimate	95% confidence interval	
0	0	0	2.47
1	0.84	0.02	4.61
2	1.71	0.21	6.04
3	2.6	0.53	7.42
4	3.51	0.95	8.77
5	4.46	1.44	10.11
6	5.43	1.99	11.47
7	6.43	2.58	12.84
8	7.46	3.22	14.23
9	8.53	3.91	15.66
10	9.64	4.63	17.12
11	10.79	5.41	18.62
12	11.99	6.22	20.17
13	13.24	7.08	21.78
14	14.54	7.99	23.46
15	15.91	8.96	25.2
16	17.35	9.98	27.04
17	18.87	11.06	28.97
18	20.47	12.21	31.01
19	22.18	13.44	33.18
20	24.02	14.75	35.52
21	25.99	16.17	38.04
22	28.14	17.69	40.8
23	30.5	19.36	43.86
24	33.13	21.2	47.3
25	36.11	23.24	51.26
26	39.57	25.55	55.98
27	43.77	28.23	61.88
28	49.19	31.46	69.93
29	57.27	35.58	82.96
30	100	44.48	100
